# Supplementary material for: Proficient brain for optimal performance: the MAP model perspective
Source: PeerJ. 2016 May 25;4:e2082. doi: 10.7717/peerj.2082 (PMC4888308; doi:10.7717/peerj.2082)

Electrode	variable
Fp1	DV1
Fpz	DV2
Fp2	DV3
F7	DV4
F3	DV5
Fz	DV6
F4	DV7
F8	DV8
FC5	DV9
FC1	DV10
FC2	DV11
FC6	DV12
T7	DV13
C3	DV14
Cz	DV15
C4	DV16
T8	DV17
CP5	DV18
CP1	DV19
CP2	DV20
CP6	DV21
P7	DV22
P3	DV23
Pz	DV24
P4	DV25
P8	DV26
POz	DV27
O1	DV28
Oz	DV29
O2	DV30

Effect	Univariate tests for repeate<br>Sigma-restricted paramete<br>Effective hypothesis decon				
	SS	Degr Free	MS	F	p
PER	2890	3	963,	5,33	0,00
Error	4873	2	180,		
TIME	112,	2	56,3	1,22	0,31
Error	827.	1	45,9		
PER*	218.	6	36,4	0,55	0,76
Error	3572	5	66,1		

	Univariate tests for repeate				
	Sigma-restricted paramete				
	Effective hypothesis decon				
Effect	SS	Degr Free	MS	F	p
PER	2148	3	716,	2,85	0,05
Error	6780	2	251,		
TIME	12,	2	6,0	0,03	0,96
Error	3111	1	172,		
PER*	322.	6	53,6	0,64	0,69
Error	4516	5	83,6		

	Univariate tests for repeate				
	Sigma-restricted paramete				
	Effective hypothesis decon				
Effect	SS	Degr Free	MS	F	p
PER	2262	3	754,	2,92	0,05
Error	6969	2	258,		
TIME	404.	2	202,	5,00	0,01
Error	727.	1	40,3		
PER*	29,	6	4,9	0,09	0,99
Error	2881	5	53,3		

	Univariate tests for repeate				
	Sigma-restricted paramete				
	Effective hypothesis decon				
Effect	SS	Degr Free	MS	F	p
PER	103.	3	34,3	0,20	0,89
Error	4571	2	169,		
TIME	195.	2	97,6	2,39	0,11
Error	734.	1	40,7		
PER*	547.	6	91,2	1,53	0,18
Error	3208	5	59,4		

	Univariate tests for repeate				
	Sigma-restricted paramete				
	Effective hypothesis decon				
Effect	SS	Degr Free	MS	F	p
PER	558.	3	186,	0,76	0,52
Error	6531	2	241,		
TIME	33,	2	16,5	0,55	0,58
Error	537.	1	29,8		
PER*	518.	6	86,3	2,30	0,04
Error	2025	5	37,5		

	Univariate tests for repeate<br>Sigma-restricted paramete<br>Effective hypothesis decon				
Effect	SS	Degr Free	MS	F	p
PER	83,	3	27,	0,24	0,86
Error	3056	2	113,		
TIME	278.	2	139,	1,43	0,26
Error	1751	1	97,2		
PER*	411,	6	68,5	1,61	0,16
Error	2296	5	42,5		

	Univariate tests for repeate<br>Sigma-restricted paramete<br>Effective hypothesis decon				
Effect	SS	Degr Free	MS	F	p
PER	883.	3	294,	2,30	0,09
Error	3442	2	127,		
TIME	492.	2	246,	2,69	0,09
Error	1642	1	91,2		
PER*	241.	6	40,2	0,41	0,86
Error	5221	5	96,7		

	Univariate tests for repeate<br>Sigma-restricted paramete<br>Effective hypothesis decon				
Effect	SS	Degr Free	MS	F	p
PER	2257	3	752,	2,12	0,12
Error	9553	2	353,		
TIME	599.	2	299,	7,31	0,00
Error	737.	1	40,9		
PER*	222.	6	37,1	0,59	0,73
Error	3358	5	62,1		

	Univariate tests for repeate<br>Sigma-restricted paramete<br>Effective hypothesis decon				
Effect	SS	Degr Free	MS	F	p
PER	531.	3	177,	0,71	0,54
Error	6659	2	246,		
TIME	280.	2	140,	5,31	0,01
Error	474.	1	26,3		
PER*	276.	6	46,0	0,86	0,52
Error	2883	5	53,4		

Effect	Univariate tests for repeate<br>Sigma-restricted paramete<br>Effective hypothesis decon				
	SS	Degr<br>Free	MS	F	p
PER	2100	3	700,:	4,23	0,01
Error	4465	2	165,:		
TIME	36,(	2	18,0	0,54	0,58
Error	592.	1	32,9		
PER*	228.	6	38,1	0,66	0,67
Error	3096	5	57,3		

Effect	Univariate tests for repeate<br>Sigma-restricted paramete<br>Effective hypothesis decon				
	SS	Degr<br>Free	MS	F	p
PER	559.	3	186,(	1,07	0,37
Error	4706	2	174,:		
TIME	151.	2	75,8	2,06	0,15
Error	662.	1	36,7		
PER*	350.	6	58,4	1,45	0,21
Error	2166	5	40,1		

Effect	Univariate tests for repeate<br>Sigma-restricted paramete<br>Effective hypothesis decon				
	SS	Degr<br>Free	MS	F	p
PER	248	3	829,	2,11	0,12
Error	1059	2	392,:		
TIME	675	2	337,	7,88	0,00
Error	771	1	42,8		
PER*	273	6	45,6	1,15	0,34
Error	213:	5	39,4		

Effect	Univariate tests for repeate<br>Sigma-restricted paramete<br>Effective hypothesis decon				
	SS	Degr<br>Free	MS	F	p
PER	467.	3	155,7	1,24	0,31
Error	3376	2	125,(		
TIME	317.	2	158,	2,71	0,09
Error	1049	1	58,3		
PER*	497.	6	82,9	1,57	0,17
Error	2840	5	52,6		

Effect	Univariate tests for repeate Sigma-restricted paramete Effective hypothesis decon				
	SS	Degr Free	MS	F	p
PER	1174	3	391,	2,35	0,09
Error	4485	2	166,		
TIME	574.	2	287,	4,05	0,03
Error	1275	1	70,8		
PER*	367.	6	61,1	1,52	0,18
Error	2172	5	40,2		

Effect	Univariate tests for repeate Sigma-restricted paramete Effective hypothesis decon				
	SS	Degr Free	MS	F	p
PER	1062	3	354,	2,51	0,07
Error	3798	2	140,		
TIME	287.	2	143,	2,15	0,14
Error	1204	1	66,9		
PER*	353.	6	58,9	1,21	0,31
Error	2611	5	48,3		

Effect	Univariate tests for repeate Sigma-restricted paramete Effective hypothesis decon				
	SS	Degr Free	MS	F	p
PER	388.	3	129,	1,13	0,35
Error	3077	2	113,		
TIME	12,	2	6,3	0,09	0,91
Error	1222	1	67,9		
PER*	357.	6	59,5	1,50	0,19
Error	2137	5	39,5		

Effect	Univariate tests for repeate Sigma-restricted paramete Effective hypothesis decon				
	SS	Degr Free	MS	F	p
PER	1111	3	370,	1,41	0,26
Error	7091	2	262,		
TIME	497.	2	248,	3,97	0,03
Error	1126	1	62,5		
PER*	244.	6	40,7	0,82	0,55
Error	2648	5	49,0		

Effect	Univariate tests for repeate<br>Sigma-restricted paramete<br>Effective hypothesis decon				
	SS	Degr<br>Free	MS	F	p
PER	207.	3	69,1	0,45	0,71
Error	4097	2	151,		
TIME	546.	2	273,	8,85	0,00
Error	555.	1	30,8		
PER*	190.	6	31,7	0,58	0,73
Error	2919	5	54,0		

Effect	Univariate tests for repeate<br>Sigma-restricted paramete<br>Effective hypothesis decon				
	SS	Degr<br>Free	MS	F	p
PER	1767	3	589,	4,05	0,01
Error	3923	2	145,		
TIME	173.	2	86,6	2,17	0,14
Error	717.	1	39,8		
PER*	300.	6	50,0	0,66	0,67
Error	4051	5	75,0		

Effect	Univariate tests for repeate<br>Sigma-restricted paramete<br>Effective hypothesis decon				
	SS	Degr<br>Free	MS	F	p
PER	848.	3	282,	3,22	0,03
Error	2370	2	87,7		
TIME	21,	2	10,6	0,16	0,84
Error	1145	1	63,6		
PER*	588.	6	98,1	2,68	0,02
Error	1977	5	36,6		

Effect	Univariate tests for repeate<br>Sigma-restricted paramete<br>Effective hypothesis decon				
	SS	Degr<br>Free	MS	F	p
PER	345.	3	115,	0,38	0,76
Error	7966	2	295,		
TIME	169.	2	84,5	1,20	0,32
Error	1267	1	70,3		
PER*	373.	6	62,2	1,62	0,15
Error	2068	5	38,2		

Effect	Univariate tests for repeate<br>Sigma-restricted paramete<br>Effective hypothesis decon				
	SS	Degr<br>Free	MS	F	p
PER	920.	3	306,	2,34	0,09
Error	3540	2	131,		
TIME	349.	2	174,	3,48	0,05
Error	904.	1	50,2		
PER*	75,	6	12,5	0,29	0,93
Error	2275	5	42,1		

Effect	Univariate tests for repeate<br>Sigma-restricted paramete<br>Effective hypothesis decon				
	SS	Degr<br>Free	MS	F	p
PER	162.	3	54,0	0,40	0,75
Error	3647	2	135,		
TIME	8,4	2	4,2	0,26	0,77
Error	286.	1	15,9		
PER*	197.	6	32,8	0,77	0,59
Error	2289	5	42,4		

Effect	Univariate tests for repeate<br>Sigma-restricted paramete<br>Effective hypothesis decon				
	SS	Degr<br>Free	MS	F	p
PER	129.	3	43,1	0,52	0,67
Error	2227	2	82,4		
TIME	19,	2	9,63	0,33	0,72
Error	521.	1	28,9		
PER*	211,	6	35,1	0,97	0,45
Error	1954	5	36,1		

Effect	Univariate tests for repeate<br>Sigma-restricted paramete<br>Effective hypothesis decon				
	SS	Degr<br>Free	MS	F	p
PER	169.	3	56,6	0,87	0,46
Error	1755	2	65,0		
TIME	123.	2	61,6	1,70	0,20
Error	649.	1	36,0		
PER*	76,	6	12,7	0,25	0,95
Error	2741	5	50,7		

Effect	Univariate tests for repeate<br>Sigma-restricted paramete<br>Effective hypothesis decon				
	SS	Degr<br>Free	MS	F	p
PER	294.	3	98,2	0,64	0,59
Error	4098	2	151,		
TIME	57,	2	28,5	1,05	0,36
Error	487.	1	27,0		
PER*	97,	6	16,2	0,32	0,91
Error	2666	5	49,3		

Effect	Univariate tests for repeate<br>Sigma-restricted paramete<br>Effective hypothesis decon				
	SS	Degr<br>Free	MS	F	p
PER	193.	3	64,6	0,32	0,80
Error	5321	2	197,		
TIME	25,	2	12,7	0,44	0,64
Error	515.	1	28,6		
PER*	106.	6	17,6	0,55	0,76
Error	1728	5	32,0		

Effect	Univariate tests for repeate<br>Sigma-restricted paramete<br>Effective hypothesis decon				
	SS	Degr<br>Free	MS	F	p
PER	231.	3	77,0	0,44	0,72
Error	4707	2	174,		
TIME	114,	2	57,2	1,57	0,23
Error	652.	1	36,2		
PER*	137.	6	22,9	0,55	0,76
Error	2244	5	41,5		

Effect	Univariate tests for repeate<br>Sigma-restricted paramete<br>Effective hypothesis decon				
	SS	Degr<br>Free	MS	F	p
PER	228.	3	76,2	0,36	0,78
Error	5709	2	211,		
TIME	129.	2	64,9	0,96	0,39
Error	1208	1	67,1		
PER*	217.	6	36,2	0,98	0,44
Error	1985	5	36,7		

Cel	LSD test; variable DV_<br>Probabilities for Post H<br>Error: Within MSE = 1				
	P	{1}<br>-6,9	{2}<br>-7,5	{3}<br>4,1	{4}<br>-7,0
1	1		0,85	0,00	0,98
2	2	0,85		0,00	0,87
3	3	0,00	0,00		0,00
4	4	0,98	0,87	0,00	

Cel	LSD test; variable DV_<br>Probabilities for Post H<br>Error: Within MSE = 1				
	P	{1}<br>-11	{2}<br>-4,9	{3}<br>-,78	{4}<br>-10
1	1		0,07	0,00	0,78
2	2	0,07		0,21	0,12
3	3	0,00	0,21		0,00
4	4	0,78	0,12	0,00	

Cel	LSD test; variable DV_<br>Probabilities for Post H<br>Error: Within MSE = 1				
	P	{1}<br>-4,2	{2}<br>-3,6	{3}<br>4,1	{4}<br>-5,6
1	1		0,83	0,01	0,64
2	2	0,83		0,01	0,50
3	3	0,01	0,01		0,00
4	4	0,64	0,50	0,00	

Cel	LSD test; variable DV_<br>Probabilities for Post H<br>Error: Within MSE = 8				
	P	{1}<br>-4,0	{2}<br>-6,3	{3}<br>,98	{4}<br>-2,9
1	1		0,35	0,04	0,65
2	2	0,35		0,00	0,17
3	3	0,04	0,00		0,11
4	4	0,65	0,17	0,11	

LSD test; variable DV_6 (medie theta i
Probabilities for Post Hoc Tests
Error: Within MSE = 37,500, df = 54,00

Ce	P	TI	{1 -10	{2 -9,	{3 -13	{4 -5,	{5 -5,	{6 -7,	{7 -7,	{8 -7,	{9 -1,	{1 -4,
1	1	1		0,8	0,2	0,1	0,0	0,2	0,3	0,3	0,0	0,0
2	1	2	0,8		0,2	0,1	0,0	0,3	0,4	0,4	0,0	0,0
3	1	3	0,2	0,2		0,0	0,0	0,0	0,0	0,0	0,0	0,0
4	2	1	0,1	0,1	0,0		0,7	0,6	0,5	0,5	0,1	0,5
5	2	2	0,0	0,0	0,0	0,7		0,4	0,3	0,3	0,2	0,7
6	2	3	0,2	0,3	0,0	0,6	0,4		0,9	0,8	0,0	0,2
7	3	1	0,3	0,4	0,0	0,5	0,3	0,9		0,9	0,0	0,2
8	3	2	0,3	0,4	0,0	0,5	0,3	0,8	0,9		0,0	0,2
9	3	3	0,0	0,0	0,0	0,1	0,2	0,0	0,0	0,0		0,3
10	4	1	0,0	0,0	0,0	0,5	0,7	0,2	0,2	0,2	0,3	
11	4	2	0,8	0,9	0,1	0,1	0,1	0,3	0,4	0,4	0,0	0,0
12	4	3	0,8	0,7	0,3	0,0	0,0	0,2	0,2	0,2	0,0	0,0

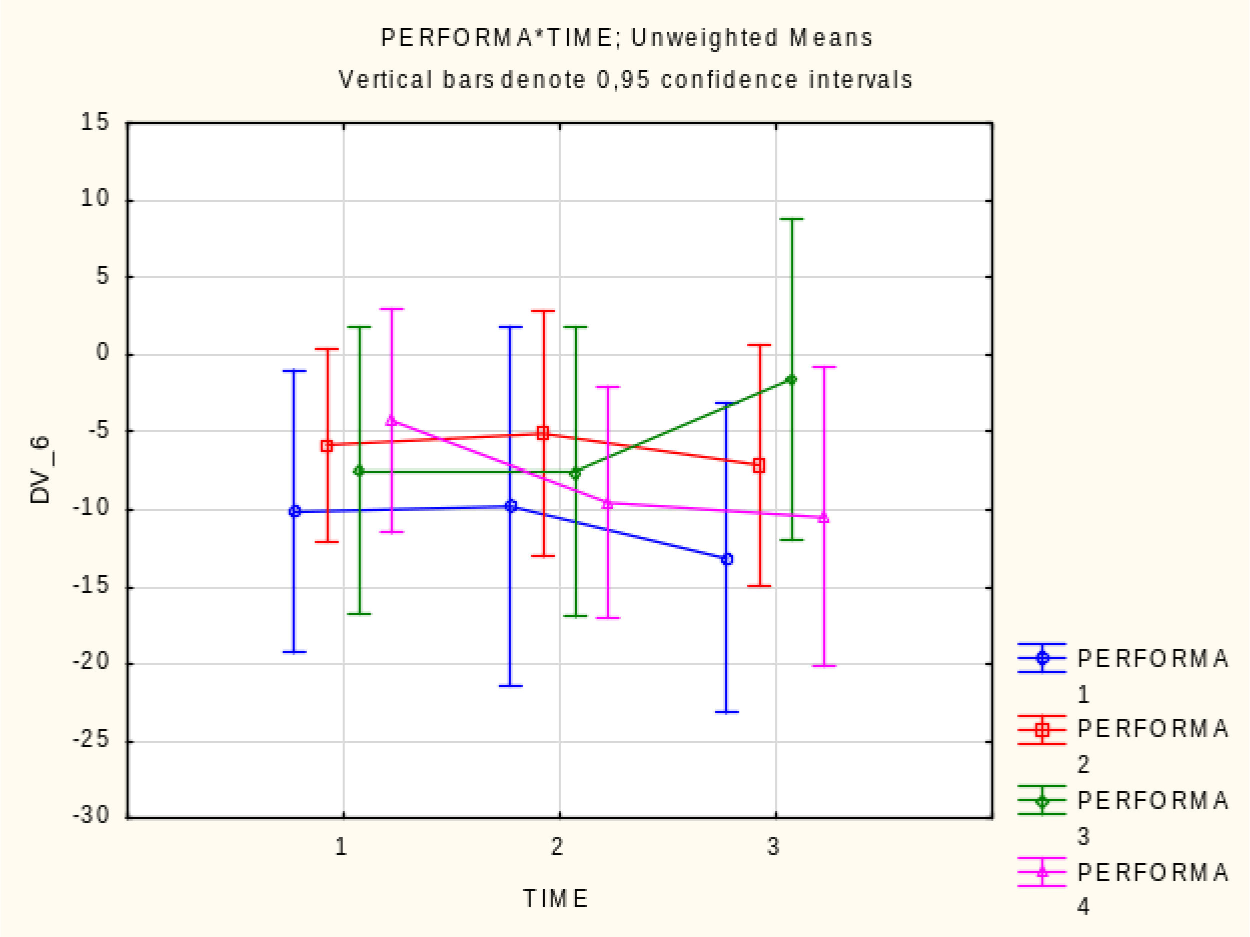

Cel	LSD test; variable DV_21 (medie theta in ana<br>Probabilities for Post Hoc Tests<br>Error: Within MSE = 36,619, df = 54,000											
	PE	TI	{1}<br>-3,0	{2}<br>-3,5	{3}<br>-5,6	{4}<br>-2,9	{5}<br>-8,0	{6}<br>-8,1	{7}<br>-1,7	{8}<br>5,1	{9}<br>-,46	{10<br>-3,0
1	1	1		0,86	0,34	0,95	0,07	0,06	0,63	0,00	0,34	0,98
2	1	2	0,86		0,43	0,82	0,10	0,09	0,52	0,00	0,26	0,88
3	1	3	0,34	0,43		0,31	0,38	0,36	0,15	0,00	0,06	0,34
4	2	1	0,95	0,82	0,31		0,06	0,05	0,67	0,00	0,37	0,94
5	2	2	0,07	0,10	0,38	0,06		0,96	0,02	0,00	0,00	0,07
6	2	3	0,06	0,09	0,36	0,05	0,96		0,02	0,00	0,00	0,06
7	3	1	0,63	0,52	0,15	0,67	0,02	0,02		0,01	0,63	0,62
8	3	2	0,00	0,00	0,00	0,00	0,00	0,00	0,01		0,04	0,00
9	3	3	0,34	0,26	0,06	0,37	0,00	0,00	0,63	0,04		0,33
10	4	1	0,98	0,88	0,34	0,94	0,07	0,06	0,62	0,00	0,33	
11	4	2	0,39	0,49	0,91	0,30	0,33	0,30	0,18	0,00	0,07	0,40
12	4	3	0,34	0,27	0,06	0,37	0,00	0,00	0,63	0,04	0,95	0,34

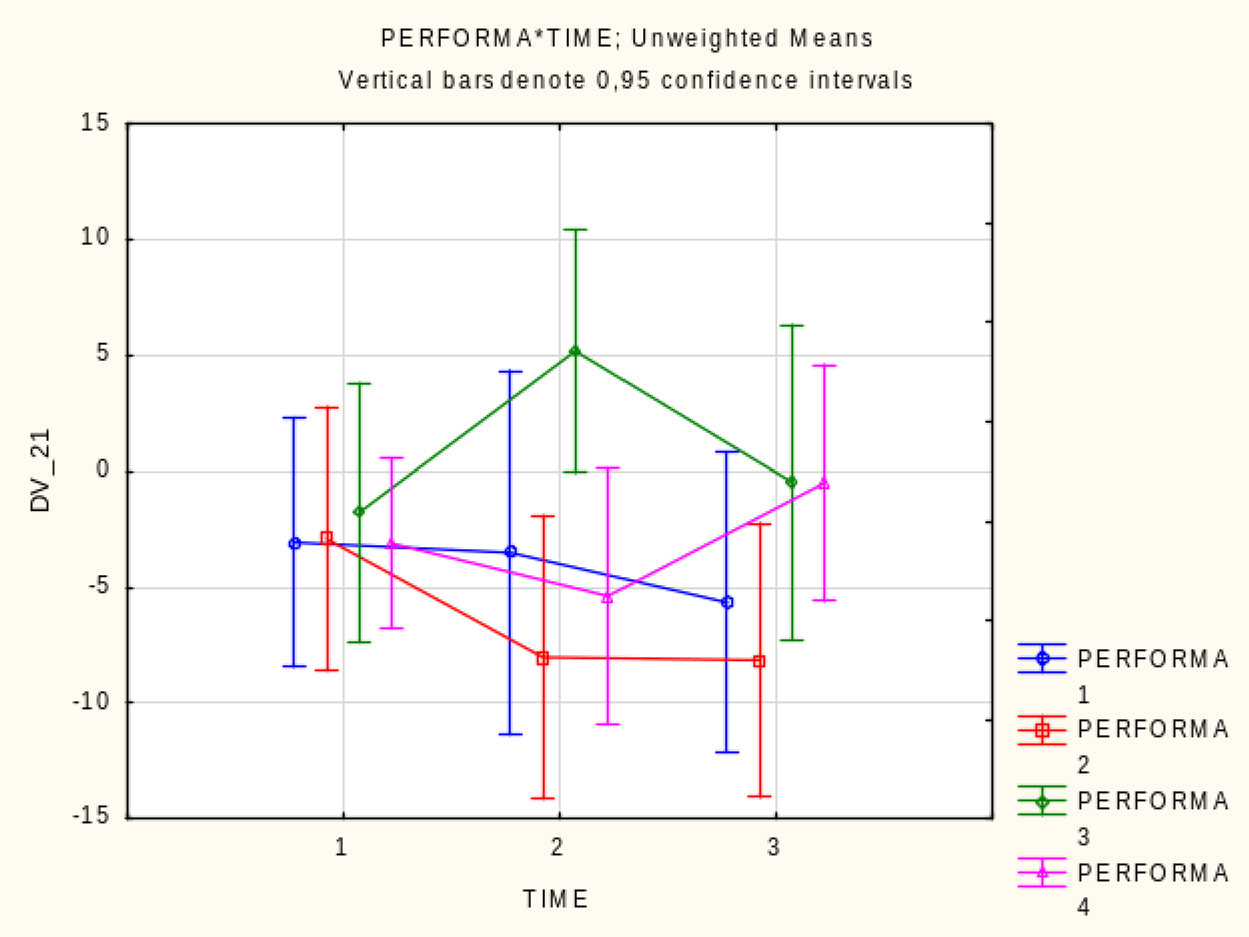

Supplement: Supplementary file 1 [file peerj-04-2082-s001.rtf]
