# Supplementary material for: Proficient brain for optimal performance: the MAP model perspective
Source: PeerJ. 2016 May 25;4:e2082. doi: 10.7717/peerj.2082 (PMC4888308; doi:10.7717/peerj.2082)
Supplement: Supplementary file 2 [file peerj-04-2082-s002.rtf]

Electrode	variable
Fp1	DV1
Fpz	DV2
Fp2	DV3
F7	DV4
F3	DV5
Fz	DV6
F4	DV7
F8	DV8
FC5	DV9
FC1	DV10
FC2	DV11
FC6	DV12
T7	DV13
C3	DV14
Cz	DV15
C4	DV16
T8	DV17
CP5	DV18
CP1	DV19
CP2	DV20
CP6	DV21
P7	DV22
P3	DV23
Pz	DV24
P4	DV25
P8	DV26
POz	DV27
O1	DV28
Oz	DV29
O2	DV30

	Univariate tests for repeate<br>Sigma-restricted paramete<br>Effective hypothesis decon				
Effect	SS	Degr Free	MS	F	p
PER	1002	3	334,	3,57	0,02
Error	2527	2	93,6		
TIME	308.	2	154,	5,46	0,01
Error	509.	1	28,2		
PER*	334.	6	55,8	2,96	0,01
Error	1015	5	18,8		

Effect	Univariate tests for repeate<br>Sigma-restricted paramete<br>Effective hypothesis decon				
	SS	Degr<br>Free	MS	F	p
PER	632.	3	210,	1,24	0,31
Error	4583	2	169,		
TIME	306.	2	153,	3,71	0,04
Error	741.	1	41,2		
PER*	130.	6	21,7	0,83	0,54
Error	1400	5	25,9		

Effect	Univariate tests for repeate<br>Sigma-restricted paramete<br>Effective hypothesis decon				
	SS	Degr<br>Free	MS	F	p
PER	102.	3	34,2	0,22	0,87
Error	4080	2	151,		
TIME	665.	2	332,	4,81	0,02
Error	1245	1	69,1		
PER*	220.	6	36,8	1,68	0,14
Error	1180	5	21,8		

Effect	Univariate tests for repeate<br>Sigma-restricted paramete<br>Effective hypothesis decon				
	SS	Degr<br>Free	MS	F	p
PER	58,	3	19,5	0,13	0,93
Error	3985	2	147,		
TIME	505.	2	252,	4,67	0,02
Error	974.	1	54,1		
PER*	135.	6	22,6	0,95	0,46
Error	1280	5	23,7		

Effect	Univariate tests for repeate<br>Sigma-restricted paramete<br>Effective hypothesis decon				
	SS	Degr<br>Free	MS	F	p
PER	254.	3	84,9	0,72	0,54
Error	3160	2	117,		
TIME	400.	2	200,	3,02	0,07
Error	1189	1	66,0		
PER*	316.	6	52,6	1,82	0,11
Error	1557	5	28,8		

Effect	Univariate tests for repeate<br>Sigma-restricted paramete<br>Effective hypothesis decon				
	SS	Degr<br>Free	MS	F	p
PER	451.	3	150,	1,07	0,37
Error	3766	2	139,		
TIME	10,	2	5,4	0,11	0,89
Error	879.	1	48,8		
PER*	309.	6	51,6	2,40	0,03
Error	1160	5	21,4		

Effect	Univariate tests for repeate<br>Sigma-restricted paramete<br>Effective hypothesis decon				
	SS	Degr<br>Free	MS	F	p
PER	70,	3	23,6	0,22	0,87
Error	2830	2	104,		
TIME	383.	2	191,	8,35	0,00
Error	413.	1	22,9		
PER*	158.	6	26,3	1,15	0,34
Error	1235	5	22,8		

Effect	Univariate tests for repeate<br>Sigma-restricted paramete<br>Effective hypothesis decon				
	SS	Degr<br>Free	MS	F	p
PER	1288	3	429,	3,03	0,04
Error	3816	2	141,		
TIME	225.	2	112,	2,21	0,13
Error	914.	1	50,7		
PER*	404.	6	67,4	3,19	0,00
Error	1140	5	21,1		

Effect	Univariate tests for repeate<br>Sigma-restricted paramete<br>Effective hypothesis decon				
	SS	Degr<br>Free	MS	F	p
PER	382.	3	127,	1,27	0,30
Error	2691	2	99,6		
TIME	344.	2	172,	8,18	0,00
Error	378.	1	21,0		
PER*	127.	6	21,3	0,78	0,58
Error	1460	5	27,0		

Effect	Univariate tests for repeate<br>Sigma-restricted paramete<br>Effective hypothesis decon				
	SS	Degr<br>Free	MS	F	p
PER	83,	3	27,8	0,29	0,82
Error	2525	2	93,5		
TIME	88,	2	44,3	1,86	0,18
Error	427.	1	23,7		
PER*	145.	6	24,2	1,18	0,32
Error	1104	5	20,4		

Effect	Univariate tests for repeate<br>Sigma-restricted paramete<br>Effective hypothesis decon				
	SS	Degr<br>Free	MS	F	p
PER	627.	3	209,	1,67	0,19
Error	3379	2	125,		
TIME	240.	2	120,	4,84	0,02
Error	447.	1	24,8		
PER*	134.	6	22,3	1,12	0,36
Error	1077	5	19,9		

Effect	Univariate tests for repeate<br>Sigma-restricted paramete<br>Effective hypothesis decon				
	SS	Degr<br>Free	MS	F	p
PER	865.	3	288,	1,52	0,23
Error	5113	2	189,		
TIME	233.	2	116,	2,41	0,11
Error	868.	1	48,2		
PER*	193.	6	32,2	1,24	0,29
Error	1396	5	25,8		

Effect	Univariate tests for repeate<br>Sigma-restricted paramete<br>Effective hypothesis decon				
	SS	Degr<br>Free	MS	F	p
PER	145	3	484,	1,04	0,38
Error	1253	2	464,		
TIME	298	2	149,	4,81	0,02
Error	557	1	30,9		
PER*	398	6	66,3	1,09	0,37
Error	326	5	60,4		

Effect	Univariate tests for repeate<br>Sigma-restricted paramete<br>Effective hypothesis decom				
	SS	Degr<br>Free	MS	F	p
PER	440.	3	146,7	2,074	0,127
Error	1910	2	70,7		
TIME	371.	2	185,5	4,689	0,022
Error	712.	1	39,5		
PER*	251.	6	41,9	2,601	0,027
Error	870.	5	16,1		

Effect	Univariate tests for repeate<br>Sigma-restricted paramete<br>Effective hypothesis decom				
	SS	Degr<br>Free	MS	F	p
PER	544.	3	181,5	1,957	0,144
Error	2503	2	92,7		
TIME	235.	2	117,5	4,322	0,029
Error	489.	1	27,1		
PER*	106.	6	17,7	0,966	0,456
Error	994.	5	18,4		

Effect	Univariate tests for repeate<br>Sigma-restricted paramete<br>Effective hypothesis decom				
	SS	Degr<br>Free	MS	F	p
PER	227.	3	75,8	0,626	0,607
Error	3302	2	122,2		
TIME	203.	2	101,7	1,284	0,302
Error	1425	1	79,2		
PER*	127.	6	21,2	0,852	0,536
Error	1349	5	24,9		

Effect	Univariate tests for repeate<br>Sigma-restricted paramete<br>Effective hypothesis decom				
	SS	Degr<br>Free	MS	F	p
PER	970.	3	323,4	1,052	0,385
Error	8305	2	307,6		
TIME	15,5	2	7,73	0,046	0,954
Error	3023	1	167,5		
PER*	474.	6	79,0	1,522	0,183
Error	2805	5	51,9		

Effect	Univariate tests for repeate<br>Sigma-restricted paramete<br>Effective hypothesis decon				
	SS	Degr<br>Free	MS	F	p
PER	762.	3	254,	1,39	0,26
Error	4916	2	182,		
TIME	252.	2	126,	3,37	0,05
Error	673.	1	37,4		
PER*	222.	6	37,0	1,13	0,35
Error	1769	5	32,7		

Effect	Univariate tests for repeate<br>Sigma-restricted paramete<br>Effective hypothesis decon				
	SS	Degr<br>Free	MS	F	p
PER	35,	3	11,8	0,11	0,95
Error	2764	2	102,		
TIME	121.	2	60,5	3,08	0,07
Error	353.	1	19,6		
PER*	278.	6	46,4	3,47	0,00
Error	721.	5	13,3		

Effect	Univariate tests for repeate<br>Sigma-restricted paramete<br>Effective hypothesis decon				
	SS	Degr<br>Free	MS	F	p
PER	872.	3	290,	2,36	0,09
Error	3326	2	123,		
TIME	36,	2	18,3	0,44	0,65
Error	749.	1	41,6		
PER*	225.	6	37,5	1,38	0,23
Error	1463	5	27,1		

Effect	Univariate tests for repeate<br>Sigma-restricted paramete<br>Effective hypothesis decon				
	SS	Degr<br>Free	MS	F	p
PER	137.	3	45,8	0,45	0,71
Error	2711	2	100,		
TIME	504.	2	252,	2,83	0,08
Error	1599	1	88,8		
PER*	178.	6	29,8	1,50	0,19
Error	1066	5	19,7		

Effect	Univariate tests for repeate<br>Sigma-restricted paramete<br>Effective hypothesis decon<br>SS	Degr<br>Free	MS	F	p
PER	1155	3	385,	1,98	0,14
Error	5240	2	194,		
TIME	266.	2	133,	2,40	0,11
Error	997.	1	55,4		
PER*	232.	6	38,6	1,23	0,30
Error	1693	5	31,3		

Effect	Univariate tests for repeate<br>Sigma-restricted paramete<br>Effective hypothesis decon<br>SS	Degr<br>Free	MS	F	p
PER	839.	3	279,	3,71	0,02
Error	2034	2	75,3		
TIME	16,	2	8,2	0,18	0,83
Error	815.	1	45,3		
PER*	61,	6	10,2	0,68	0,66
Error	813.	5	15,0		

Effect	Univariate tests for repeate<br>Sigma-restricted paramete<br>Effective hypothesis decon<br>SS	Degr<br>Free	MS	F	p
PER	319.	3	106,	0,71	0,55
Error	4050	2	150,		
TIME	6,2	2	3,1	0,06	0,93
Error	837.	1	46,5		
PER*	230.	6	38,3	1,71	0,13
Error	1211	5	22,4		

Effect	Univariate tests for repeate<br>Sigma-restricted paramete<br>Effective hypothesis decon<br>SS	Degr<br>Free	MS	F	p
PER	581.	3	193,	1,46	0,24
Error	3568	2	132,		
TIME	68,	2	34,0	0,80	0,46
Error	764.	1	42,4		
PER*	151.	6	25,2	0,88	0,51
Error	1538	5	28,4		

Effect	SS	Degr Free	MS	F	p
	Univariate tests for repeate Sigma-restricted paramete Effective hypothesis decon				
PER	472.	3	157,	2,16	0,11
Error	1964	2	72,7		
TIME	56,	2	28,2	0,96	0,39
Error	525.	1	29,1		
PER*	152.	6	25,3	1,11	0,36
Error	1228	5	22,7		

Effect	SS	Degr Free	MS	F	p
	Univariate tests for repeate Sigma-restricted paramete Effective hypothesis decon				
PER	348.	3	116,	1,11	0,35
Error	2808	2	104,		
TIME	115,	2	57,7	1,05	0,36
Error	985.	1	54,7		
PER*	145.	6	24,2	0,80	0,57
Error	1624	5	30,0		

Effect	SS	Degr Free	MS	F	p
	Univariate tests for repeate Sigma-restricted paramete Effective hypothesis decon				
PER	740.	3	246,	1,29	0,29
Error	5145	2	190,		
TIME	27,	2	13,5	0,26	0,76
Error	916.	1	50,8		
PER*	136.	6	22,7	0,78	0,58
Error	1567	5	29,0		

Effect	SS	Degr Free	MS	F	p
	Univariate tests for repeate Sigma-restricted paramete Effective hypothesis decon				
PER	552.	3	184,	1,38	0,26
Error	3597	2	133,		
TIME	428.	2	214,	2,87	0,08
Error	1342	1	74,5		
PER*	163.	6	27,2	1,12	0,35
Error	1304	5	24,1		

Effect	Univariate tests for repeate<br>Sigma-restricted paramete<br>Effective hypothesis decon				
	SS	Degr<br>Free	MS	F	p
PER	61,	3	20,4	0,11	0,95
Error	4800	2	177,		
TIME	794.	2	397,	3,53	0,05
Error	2024	1	112,		
PER*	279.	6	46,6	1,00	0,42
Error	2494	5	46,1		

Cel	LSD test; variable DV_<br>Probabilities for Post H<br>Error: Within MSE = 9				
	PE	{1}<br>1,4	{2}<br>-5,7	{3}<br>-3,7	{4}<br>-5,3
1	1		0,00	0,04	0,01
2	2	0,00		0,43	0,87
3	3	0,04	0,43		0,53
4	4	0,01	0,87	0,53	

Cel	LSD test; variable DV_<br>Probabilities for Post H<br>Error: Within MSE = 1				
	PE	{1}<br>,35	{2}<br>-3,	{3}<br>-2,5	{4}<br>-8,6
1	1		0,26	0,35	0,00
2	2	0,26		0,85	0,08
3	3	0,35	0,85		0,05
4	4	0,00	0,08	0,05	

Cel	LSD test; variable DV_<br>Probabilities for Post H<br>Error: Within MSE = 7				
	PE	{1}<br>6,5	{2}<br>-,15	{3}<br>1,2	{4}<br>,45
1	1		0,00	0,02	0,01
2	2	0,00		0,54	0,78
3	3	0,02	0,54		0,73
4	4	0,01	0,78	0,73	

Cell	PI	TI	LSD test; variable DV_1 (medie high alpha in a<br>Probabilities for Post Hoc Tests<br>Error: Within MSE = 18,807, df = 54,000<br>{1}<br>-,80	{2}<br>3,3	{3}<br>1,9	{4}<br>-3,5	{5}<br>-3,3	{6}<br>-10	{7}<br>-1,1	{8}<br>-3,3	{9}<br>-6,8	{10<br>-3,3
1	1	1		0,03	0,16	0,15	0,19	0,00	0,84	0,19	0,00	0,19
2	1	2	0,03		0,48	0,00	0,00	0,00	0,02	0,00	0,00	0,00
3	1	3	0,16	0,48		0,00	0,00	0,00	0,11	0,00	0,00	0,00
4	2	1	0,15	0,00	0,00		0,91	0,00	0,22	0,89	0,09	0,90
5	2	2	0,19	0,00	0,00	0,91		0,00	0,26	0,98	0,07	0,99
6	2	3	0,00	0,00	0,00	0,00	0,00		0,00	0,00	0,07	0,00
7	3	1	0,84	0,02	0,11	0,22	0,26	0,00		0,27	0,00	0,26
8	3	2	0,19	0,00	0,00	0,89	0,98	0,00	0,27		0,07	0,98
9	3	3	0,00	0,00	0,00	0,09	0,07	0,07	0,00	0,07		0,07
10	4	1	0,19	0,00	0,00	0,90	0,99	0,00	0,26	0,98	0,07	
11	4	2	0,01	0,00	0,00	0,33	0,28	0,01	0,03	0,27	0,48	0,28
12	4	3	0,00	0,00	0,00	0,06	0,04	0,11	0,00	0,04	0,82	0,04

Cell	PE	TII	LSD test; variable DV_6 (medie high alpha in anali<br>Probabilities for Post Hoc Tests<br>Error: Within MSE = 21,494, df = 54,000<br>{1}<br>-6,7	{2}<br>-5,5	{3}<br>-2,8	{4}<br>-1,6	{5}<br>,402	{6}<br>-1,7	{7}<br>-6,2	{8}<br>-2,0	{9}<br>-5,7	{10<br>-3,6
1	1	1		0,56	0,06	0,01	0,00	0,01	0,81	0,02	0,64	0,13
2	1	2	0,56		0,19	0,06	0,00	0,06	0,73	0,09	0,91	0,35
3	1	3	0,06	0,19		0,56	0,12	0,58	0,10	0,70	0,16	0,71
4	2	1	0,01	0,06	0,56		0,33	0,97	0,02	0,84	0,05	0,34
5	2	2	0,00	0,00	0,12	0,33		0,31	0,00	0,24	0,00	0,05
6	2	3	0,01	0,06	0,58	0,97	0,31		0,03	0,86	0,05	0,36
7	3	1	0,81	0,73	0,10	0,02	0,00	0,03		0,04	0,82	0,20
8	3	2	0,02	0,09	0,70	0,84	0,24	0,86	0,04		0,07	0,45
9	3	3	0,64	0,91	0,16	0,05	0,00	0,05	0,82	0,07		0,29
10	4	1	0,13	0,35	0,71	0,34	0,05	0,36	0,20	0,45	0,29	
11	4	2	0,51	0,22	0,01	0,00	0,00	0,00	0,37	0,00	0,26	0,03
12	4	3	0,96	0,59	0,07	0,01	0,00	0,02	0,84	0,03	0,67	0,14

Cell	LSD test; variable DV_8 (medie high alpha in analis<br>Probabilities for Post Hoc Tests<br>Error: Within MSE = 21,113, df = 54,000<br>PE	TII	{1}<br>-,79	{2}<br>,376	{3}<br>1,49	{4}<br>-,06	{5}<br>-2,7	{6}<br>-6,5	{7}<br>-1,3	{8}<br>,747	{9}<br>-7,0	{10<br>-6,7
1	1	1		0,57	0,26	0,72	0,34	0,00	0,79	0,45	0,00	0,00
2	1	2	0,57		0,58	0,83	0,13	0,00	0,41	0,85	0,00	0,00
3	1	3	0,26	0,58		0,45	0,04	0,00	0,17	0,71	0,00	0,00
4	2	1	0,72	0,83	0,45		0,19	0,00	0,54	0,69	0,00	0,00
5	2	2	0,34	0,13	0,04	0,19		0,06	0,48	0,09	0,03	0,05
6	2	3	0,00	0,00	0,00	0,00	0,06		0,01	0,00	0,80	0,94
7	3	1	0,79	0,41	0,17	0,54	0,48	0,01		0,31	0,00	0,01
8	3	2	0,45	0,85	0,71	0,69	0,09	0,00	0,31		0,00	0,00
9	3	3	0,00	0,00	0,00	0,00	0,03	0,80	0,00	0,00		0,85
10	4	1	0,00	0,00	0,00	0,00	0,05	0,94	0,01	0,00	0,85	
11	4	2	0,00	0,00	0,00	0,00	0,00	0,10	0,00	0,00	0,17	0,12
12	4	3	0,00	0,00	0,00	0,00	0,00	0,16	0,00	0,00	0,25	0,18

Cel	LSD test; variable DV_14 (medie high alpha<br>Probabilities for Post Hoc Tests<br>Error: Within MSE = 16,113, df = 54,000<br>PE	TI	{1}<br>,49	{2}<br>2,2	{3}<br>-1,	{4}<br>1,7	{5}<br>,90	{6}<br>-4,	{7}<br>,92	{8}<br>-,0	{9}<br>-5,	{10<br>-2,
1	1	1		0,3	0,3	0,4	0,8	0,0	0,8	0,7	0,0	0,0
2	1	2	0,3		0,0	0,8	0,4	0,0	0,4	0,2	0,0	0,0
3	1	3	0,3	0,0		0,0	0,2	0,0	0,2	0,5	0,0	0,3
4	2	1	0,4	0,8	0,0		0,6	0,0	0,6	0,3	0,0	0,0
5	2	2	0,8	0,4	0,2	0,6		0,0	0,9	0,5	0,0	0,0
6	2	3	0,0	0,0	0,0	0,0	0,0		0,0	0,0	0,5	0,3
7	3	1	0,8	0,4	0,2	0,6	0,9	0,0		0,5	0,0	0,0
8	3	2	0,7	0,2	0,5	0,3	0,5	0,0	0,5		0,0	0,1
9	3	3	0,0	0,0	0,0	0,0	0,0	0,5	0,0	0,0		0,11
10	4	1	0,0	0,0	0,3	0,0	0,0	0,3	0,0	0,1	0,11	
11	4	2	0,0	0,0	0,0	0,0	0,0	0,2	0,0	0,0	0,6	0,0
12	4	3	0,0	0,0	0,0	0,0	0,0	0,9	0,0	0,0	0,5	0,3

LSD test; variable DV_19 (medie high alpha
Probabilities for Post Hoc Tests
Error: Within MSE = 13,364, df = 54,000

Cel	PI	TI	{1} 2,4	{2} -,2	{3} -1,	{4} ,27	{5} 2,5	{6} -3,	{7} 2,7	{8} 1,6	{9} -2,	{10 -1,
1	1	1		0,1	0,0	0,1	0,9	0,0	0,8	0,6	0,0	0,0
2	1	2	0,1		0,6	0,7	0,0	0,0	0,0	0,2	0,2	0,3
3	1	3	0,0	0,6		0,4	0,0	0,2	0,0	0,1	0,5	0,5
4	2	1	0,1	0,7	0,4		0,1	0,0	0,1	0,4	0,1	0,1
5	2	2	0,9	0,0	0,0	0,1		0,0	0,9	0,5	0,0	0,0
6	2	3	0,0	0,0	0,2	0,0	0,0		0,0	0,0	0,5	0,4
7	3	1	0,8	0,0	0,0	0,1	0,9	0,0		0,4	0,0	0,0
8	3	2	0,6	0,2	0,1	0,4	0,5	0,0	0,4		0,0	0,0
9	3	3	0,0	0,2	0,5	0,1	0,0	0,5	0,0	0,0		0,9
10	4	1	0,0	0,3	0,5	0,1	0,0	0,4	0,0	0,0	0,9	
11	4	2	0,0	0,6	0,9	0,4	0,0	0,2	0,0	0,1	0,5	0,5
12	4	3	0,3	0,4	0,2	0,6	0,3	0,0	0,2	0,6	0,0	0,0

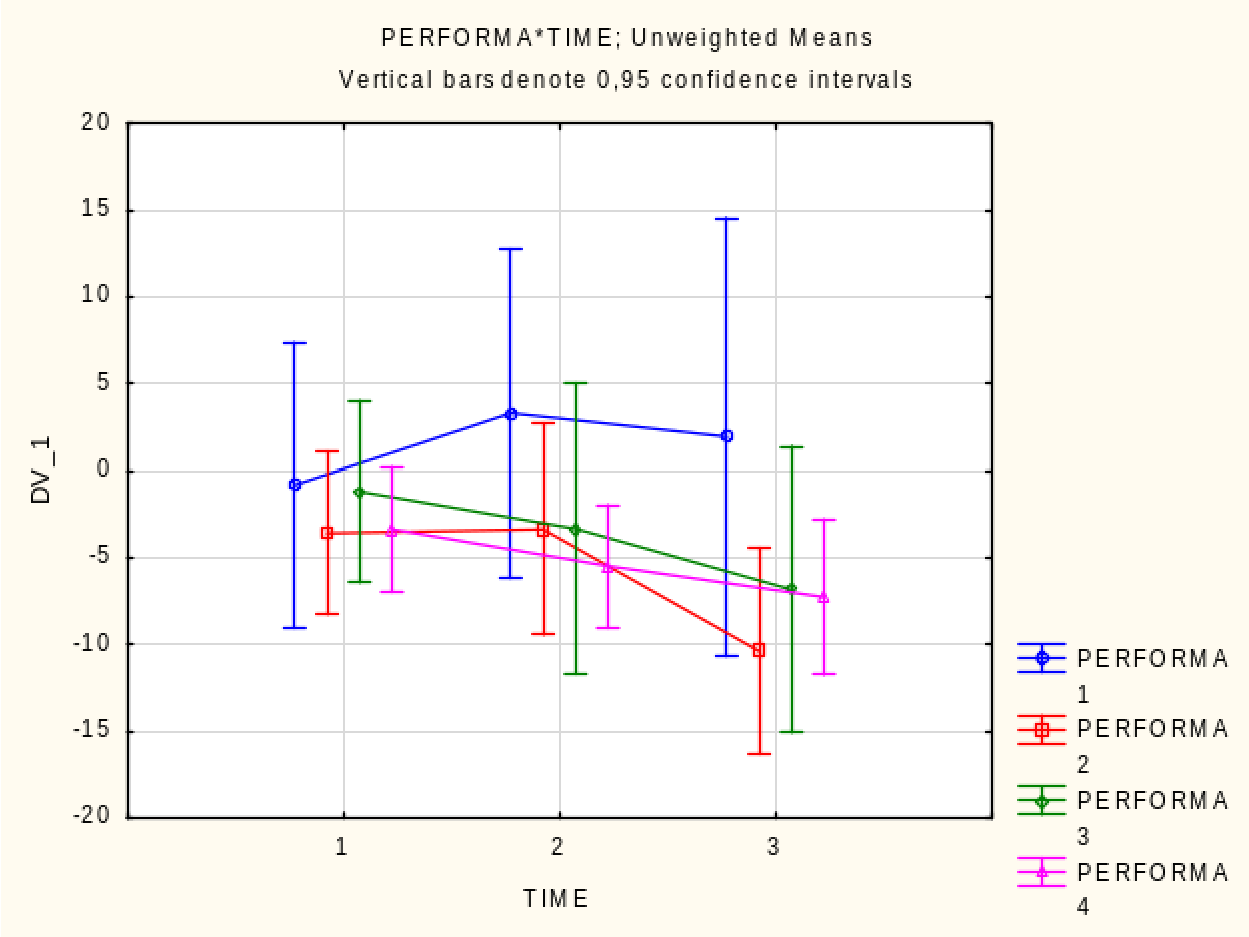

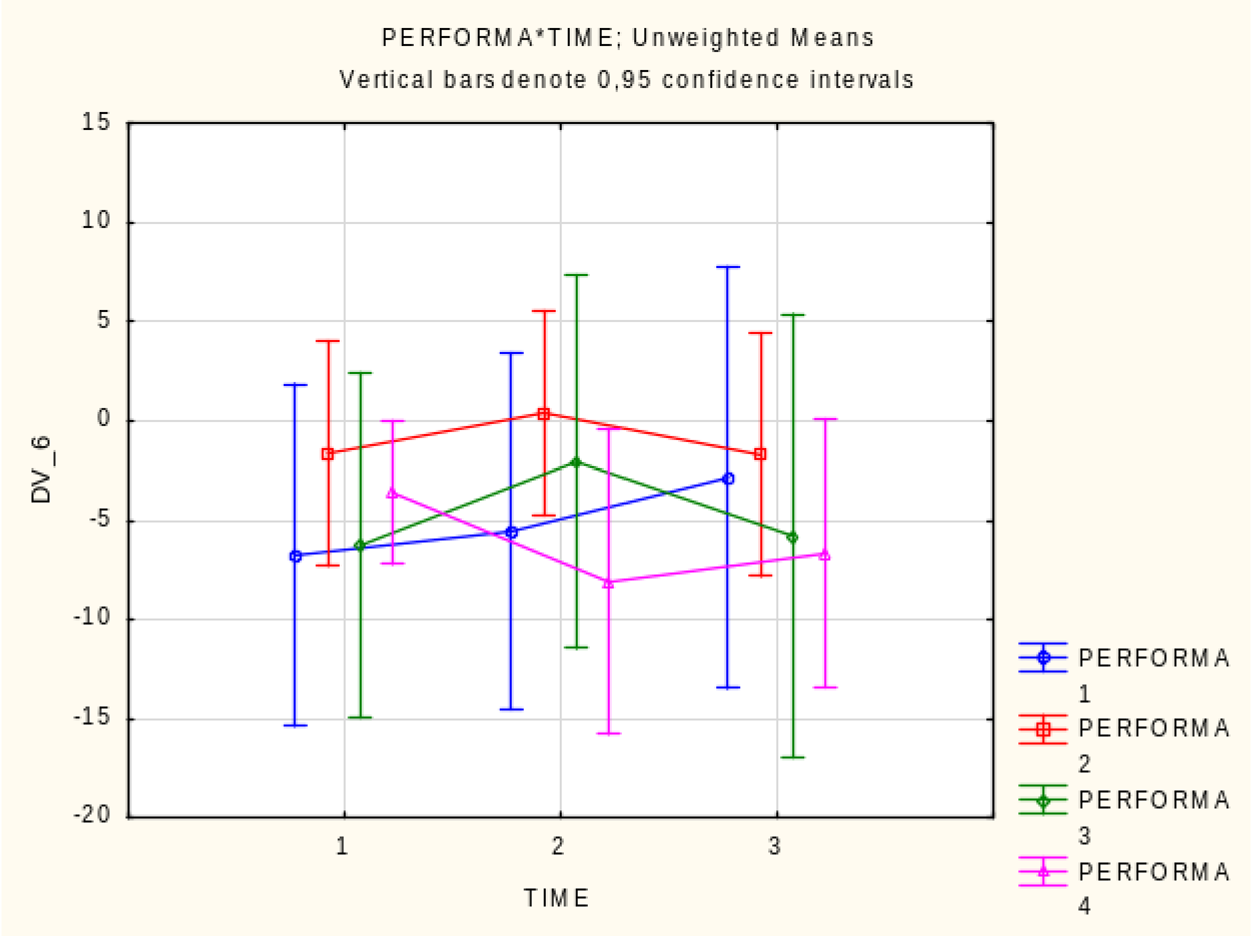
PERFORMA*TIME; Unweighted Means
Vertical bars denote 0,95 confidence intervals
15
10
5
0
-5
-10
-15
-20
DV_6
1
2
3
TIME
PERFORMA 1
PERFORMA 2
PERFORMA 3
PERFORMA 4

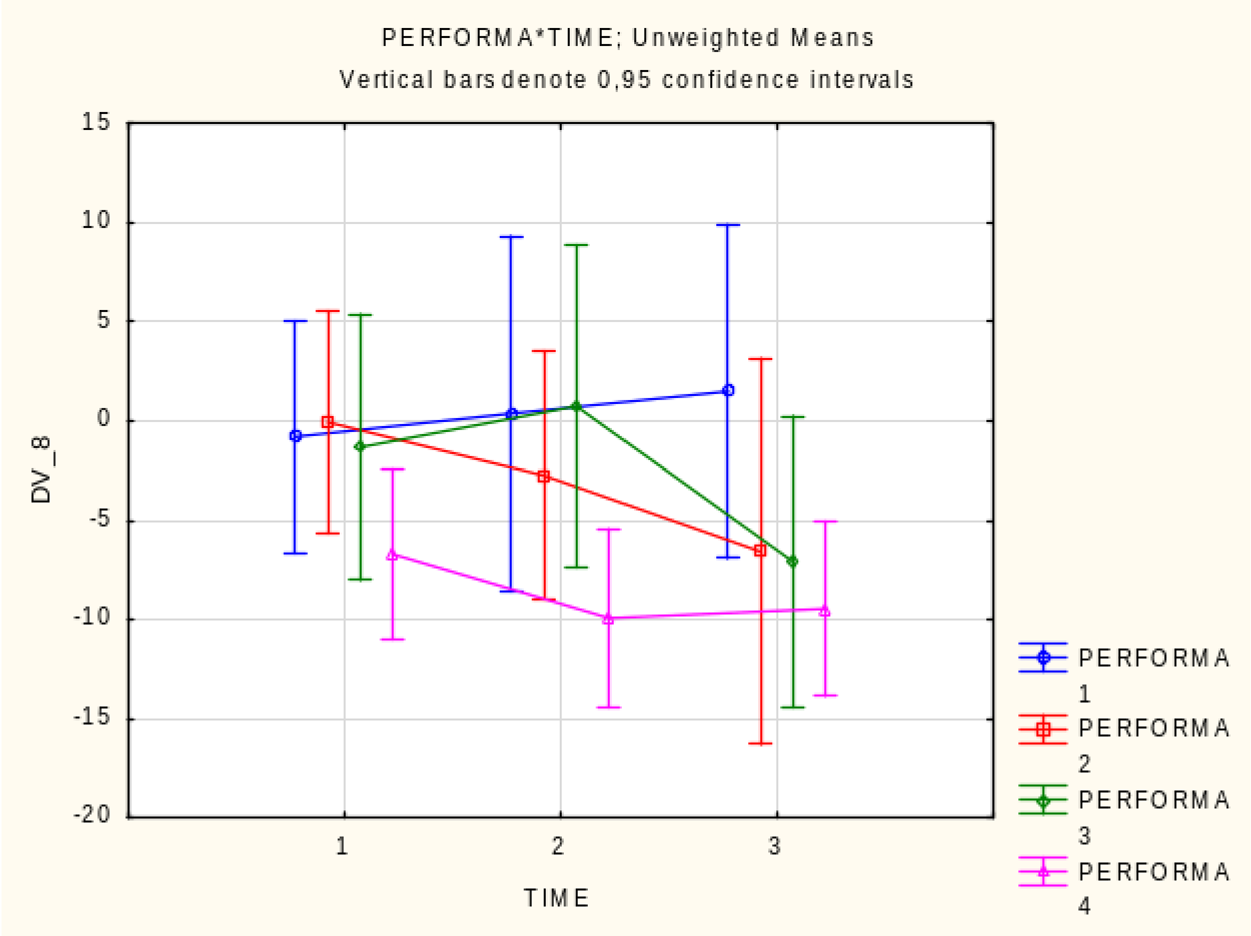

PERFORMA*TIME; Unweighted Means
Vertical bars denote 0,95 confidence intervals
15
10
5
0
-5
-10
-15
-20
DV_8
1
2
3
TIME
PERFORMA 1
PERFORMA 2
PERFORMA 3
PERFORMA 4

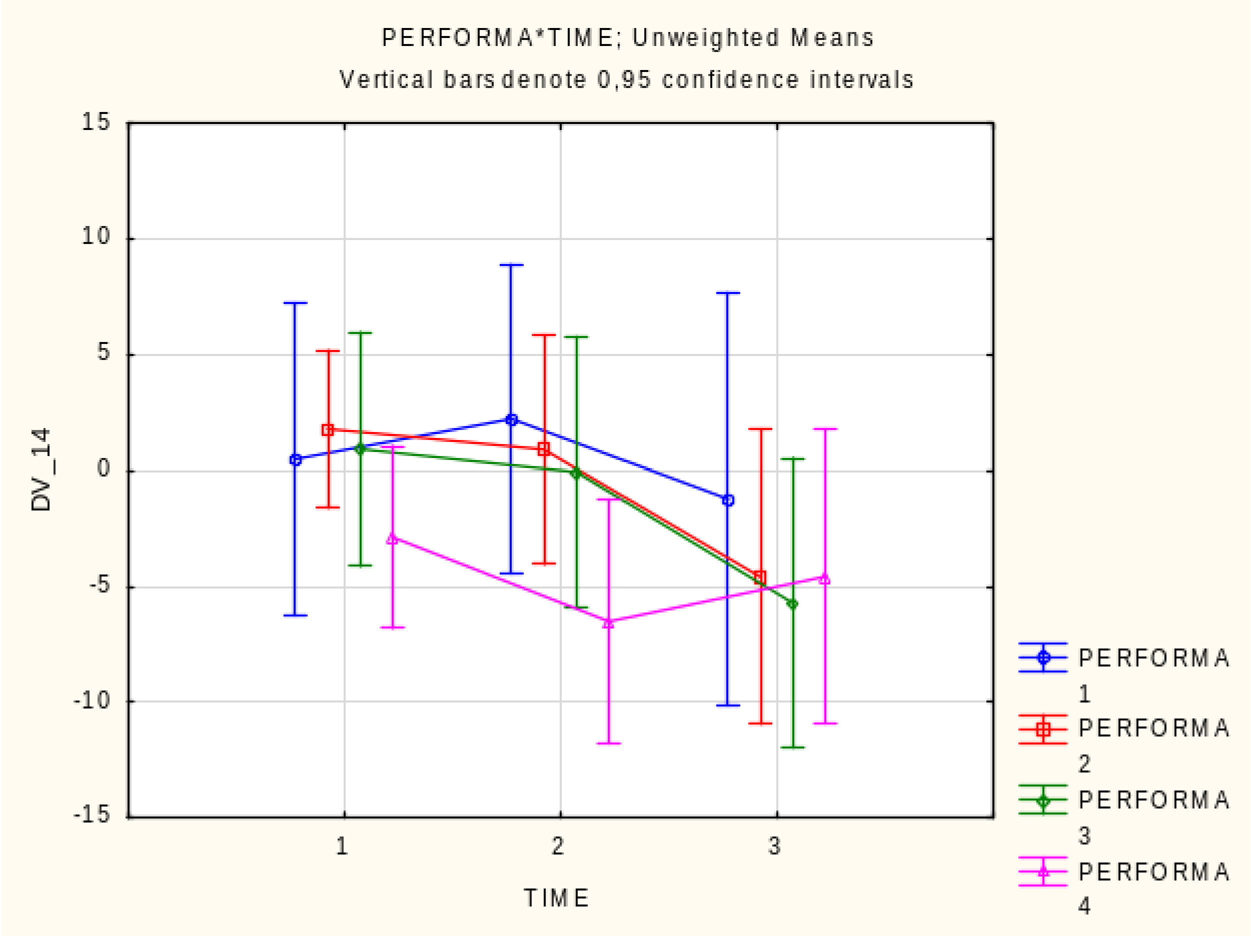

PERFORMA*TIME; Unweighted Means
Vertical bars denote 0,95 confidence intervals
15
10
5
0
-5
-10
-15
DV_14
1
2
3
TIME
PERFORMA 1
PERFORMA 2
PERFORMA 3
PERFORMA 4

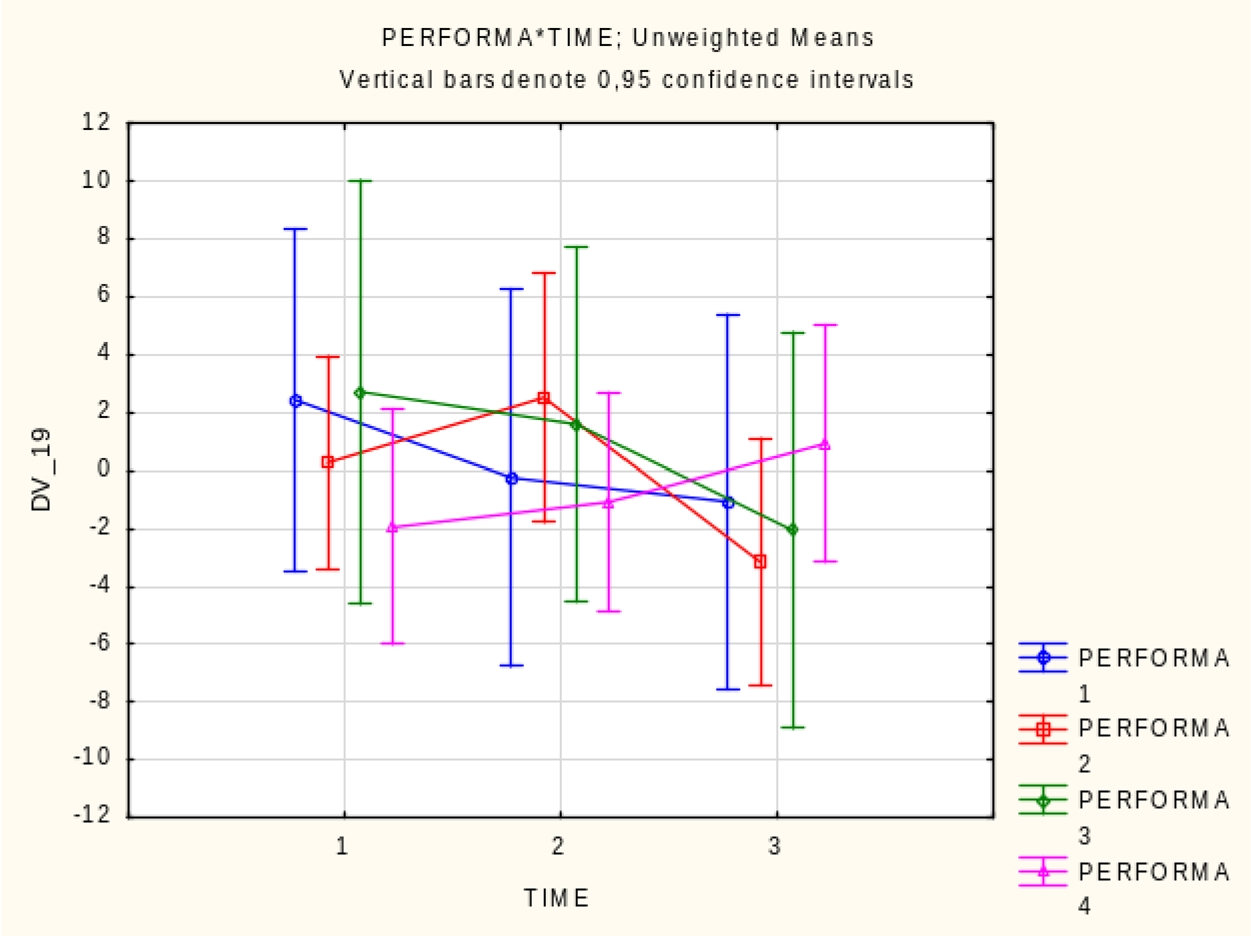
PERFORMA*TIME; Unweighted Means
Vertical bars denote 0,95 confidence intervals
DV_19
12
10
8
6
4
2
0
-2
-4
-6
-8
-10
-12
1
2
3
TIME
PERFORMA 1
PERFORMA 2
PERFORMA 3
PERFORMA 4
