# Supplementary material for: Proficient brain for optimal performance: the MAP model perspective
Source: PeerJ. 2016 May 25;4:e2082. doi: 10.7717/peerj.2082 (PMC4888308; doi:10.7717/peerj.2082)
Supplement: Supplementary file 3 [file peerj-04-2082-s003.rtf]

Electrode	variable
Fp1	DV1
Fpz	DV2
Fp2	DV3
F7	DV4
F3	DV5
Fz	DV6
F4	DV7
F8	DV8
FC5	DV9
FC1	DV10
FC2	DV11
FC6	DV12
T7	DV13
C3	DV14
Cz	DV15
C4	DV16
T8	DV17
CP5	DV18
CP1	DV19
CP2	DV20
CP6	DV21
P7	DV22
P3	DV23
Pz	DV24
P4	DV25
P8	DV26
POz	DV27
O1	DV28
Oz	DV29
O2	DV30

	Univariate tests for repeate<br>Sigma-restricted paramete<br>Effective hypothesis decon				
Effect	SS	Degr Free	MS	F	p
PER	613.	3	204,	1,35	0,27
Error	4065	2	150,		
TIME	437.	2	218,	3,96	0,03
Error	991.	1	55,0		
PER*	336.	6	56,1	1,46	0,20
Error	2064	5	38,2		

	Univariate tests for repeate<br>Sigma-restricted paramete<br>Effective hypothesis decon				
Effect	SS	Degr<br>Free	MS	F	p
PER	613.	3	204,	1,35	0,27
Error	4065	2	150,		
TIME	437.	2	218,	3,96	0,03
Error	991.	1	55,0		
PER*	336.	6	56,1	1,46	0,20
Error	2064	5	38,2		

	Univariate tests for repeate<br>Sigma-restricted paramete<br>Effective hypothesis decon				
Effect	SS	Degr<br>Free	MS	F	p
PER	1511	3	503,	1,59	0,21
Error	8514	2	315,		
TIME	285.	2	142,	4,63	0,02
Error	553.	1	30,7		
PER*	48,	6	8,1	0,14	0,98
Error	3007	5	55,6		

	Univariate tests for repeate<br>Sigma-restricted paramete<br>Effective hypothesis decon				
Effect	SS	Degr<br>Free	MS	F	p
PER	374.	3	124,	0,69	0,56
Error	4827	2	178,		
TIME	767.	2	383,	8,90	0,00
Error	775.	1	43,1		
PER*	92,	6	15,4	0,45	0,84
Error	1842	5	34,1		

	Univariate tests for repeate<br>Sigma-restricted paramete<br>Effective hypothesis decon				
Effect	SS	Degr<br>Free	MS	F	p
PER	793.	3	264,	2,21	0,10
Error	3227	2	119,		
TIME	510.	2	255,	3,76	0,04
Error	1219	1	67,7		
PER*	483.	6	80,6	1,95	0,08
Error	2229	5	41,2		

Effect	Univariate tests for repeate<br>Sigma-restricted paramete<br>Effective hypothesis decon				
	SS	Degr<br>Free	MS	F	p
PER	463.	3	154,	1,94	0,14
Error	2140	2	79,2		
TIME	378.	2	189,	8,80	0,00
Error	386.	1	21,4		
PER*	289.	6	48,2	1,48	0,20
Error	1753	5	32,4		

Effect	Univariate tests for repeate<br>Sigma-restricted paramete<br>Effective hypothesis decon				
	SS	Degr<br>Free	MS	F	p
PER	1589	3	529,	3,17	0,04
Error	4511	2	167,		
TIME	458.	2	229,	3,84	0,04
Error	1072	1	59,5		
PER*	409.	6	68,2	1,28	0,27
Error	2863	5	53,0		

Effect	Univariate tests for repeate<br>Sigma-restricted paramete<br>Effective hypothesis decon				
	SS	Degr<br>Free	MS	F	p
PER	1489	3	496,	2,93	0,05
Error	4564	2	169,		
TIME	571.	2	285,	8,87	0,00
Error	579.	1	32,2		
PER*	187.	6	31,2	0,69	0,65
Error	2415	5	44,7		

Effect	Univariate tests for repeate<br>Sigma-restricted paramete<br>Effective hypothesis decon				
	SS	Degr<br>Free	MS	F	p
PER	461.	3	153,	1,28	0,29
Error	3236	2	119,8		
TIME	192.	2	96,1	4,50	0,02
Error	384.	1	21,3		
PER*	65,8	6	10,9	0,32	0,92
Error	1828	5	33,8		

Effect	Univariate tests for repeate<br>Sigma-restricted paramete<br>Effective hypothesis decon				
	SS	Degr<br>Free	MS	F	p
PER	1759	3	586,	5,01	0,00
Error	3157	2	116,		
TIME	159.	2	79,8	2,24	0,13
Error	640.	1	35,5		
PER*	39,	6	6,5	0,21	0,97
Error	1669	5	30,9		

Effect	Univariate tests for repeate<br>Sigma-restricted paramete<br>Effective hypothesis decon				
	SS	Degr<br>Free	MS	F	p
PER	842	3	280,	0,50	0,68
Error	1512	2	560,		
TIME	803	2	401,	5,54	0,01
Error	130	1	72,4		
PER*	46,	6	7,7	0,11	0,99
Error	376	5	69,7		

Effect	Univariate tests for repeate<br>Sigma-restricted paramete<br>Effective hypothesis decon				
	SS	Degr<br>Free	MS	F	p
PER	364.	3	121,	0,94	0,43
Error	3457	2	128,		
TIME	491.	2	245,	9,10	0,00
Error	485.	1	26,9		
PER*	159.	6	26,6	1,03	0,41
Error	1389	5	25,7		

Effect	Univariate tests for repeate<br>Sigma-restricted paramete<br>Effective hypothesis decon				
	SS	Degr<br>Free	MS	F	p
PER	704.	3	234,	1,71	0,18
Error	3704	2	137,		
TIME	597.	2	298,	6,12	0,00
Error	877.	1	48,7		
PER*	122.	6	20,4	0,55	0,76
Error	1978	5	36,6		

Effect	Univariate tests for repeate<br>Sigma-restricted paramete<br>Effective hypothesis decon				
	SS	Degr<br>Free	MS	F	p
PER	477.	3	159,	0,75	0,52
Error	5700	2	211,		
TIME	532.	2	266,	6,19	0,00
Error	773.	1	42,9		
PER*	333.	6	55,5	1,02	0,41
Error	2919	5	54,0		

Effect	Univariate tests for repeate<br>Sigma-restricted paramete<br>Effective hypothesis decon				
	SS	Degr<br>Free	MS	F	p
PER	141.	3	47,1	0,37	0,77
Error	3395	2	125,		
TIME	280.	2	140,	10,9	0,00
Error	231.	1	12,8		
PER*	222.	6	37,1	1,20	0,32
Error	1669	5	30,9		

Effect	Univariate tests for repeate<br>Sigma-restricted paramete<br>Effective hypothesis decon				
	SS	Degr<br>Free	MS	F	p
PER	211,	3	70,5	0,34	0,79
Error	5520	2	204,		
TIME	487.	2	243,	5,84	0,01
Error	750.	1	41,7		
PER*	81,	6	13,6	0,33	0,91
Error	2179	5	40,3		

Effect	Univariate tests for repeate<br>Sigma-restricted paramete<br>Effective hypothesis decon				
	SS	Degr<br>Free	MS	F	p
PER	17,	3	5,6	0,02	0,99
Error	6499	2	240,		
TIME	597.	2	298,	5,55	0,01
Error	968.	1	53,8		
PER*	160.	6	26,7	0,58	0,73
Error	2458	5	45,5		

LSD test; variable DV_
Probabilities for Post H
Error: Within MSE = 1

Cel	PE	{1} -1,4	{2} -3,9	{3} -3,0	{4} -10
1	1		0,44	0,63	0,00
2	2	0,44		0,77	0,04
3	3	0,63	0,77		0,02
4	4	0,00	0,04	0,02	

LSD test; variable DV_
Probabilities for Post H
Error: Within MSE = 1

Cel	PE	{1} -9,1	{2} -2,7	{3} 1,1	{4} -6,1
1	1		0,03	0,00	0,29
2	2	0,03		0,17	0,23
3	3	0,00	0,17		0,01
4	4	0,29	0,23	0,01	
